# Success Factors of European Syndromic Surveillance Systems: A Worked Example of Applying Qualitative Comparative Analysis

**DOI:** 10.1371/journal.pone.0155535

**Published:** 2016-05-16

**Authors:** Alexandra Ziemann, Anne Fouillet, Helmut Brand, Thomas Krafft

**Affiliations:** 1 Department of International Health, CAPHRI School of Public Health and Primary Care, Faculty of Health, Medicine and Life Sciences, Maastricht University, Maastricht, The Netherlands; 2 Institute de Veille Sanitaire, Saint Maurice cedex, France; 3 Department of Health, Ethics and Society, CAPHRI School of Public Health and Primary Care, Faculty of Health, Medicine and Life Sciences, Maastricht University, Maastricht, The Netherlands; Institut Pasteur, FRANCE

## Abstract

**Introduction:**

Syndromic surveillance aims at augmenting traditional public health surveillance with timely information. To gain a head start, it mainly analyses existing data such as from web searches or patient records. Despite the setup of many syndromic surveillance systems, there is still much doubt about the benefit of the approach. There are diverse interactions between performance indicators such as timeliness and various system characteristics. This makes the performance assessment of syndromic surveillance systems a complex endeavour. We assessed if the comparison of several syndromic surveillance systems through Qualitative Comparative Analysis helps to evaluate performance and identify key success factors.

**Materials and Methods:**

We compiled case-based, mixed data on performance and characteristics of 19 syndromic surveillance systems in Europe from scientific and grey literature and from site visits. We identified success factors by applying crisp-set Qualitative Comparative Analysis. We focused on two main areas of syndromic surveillance application: seasonal influenza surveillance and situational awareness during different types of potentially health threatening events.

**Results:**

We found that syndromic surveillance systems might detect the onset or peak of seasonal influenza earlier if they analyse non-clinical data sources. Timely situational awareness during different types of events is supported by an automated syndromic surveillance system capable of analysing multiple syndromes. To our surprise, the analysis of multiple data sources was no key success factor for situational awareness.

**Conclusions:**

We suggest to consider these key success factors when designing or further developing syndromic surveillance systems. Qualitative Comparative Analysis helped interpreting complex, mixed data on small-N cases and resulted in concrete and practically relevant findings.

## Introduction

Syndromic surveillance aims at augmenting traditional public health surveillance systems by providing (near) real-time information on the public health impact of events. To gain a head start, syndromic surveillance mainly analyses syndromes generated from existing data that originally were not collected for surveillance purposes [1]. Such data can come from web query logs, telephone helpline registries, or patient or veterinary records [2]. Syndromic surveillance is used for two main purposes. The first is timely infectious disease outbreak detection. The second purpose is near real-time situational awareness during events such as mass gatherings or extreme weather events [3]. Over the past 15 years, an increasing number of syndromic surveillance systems were set up in Europe and elsewhere. There is still doubt about the benefit of the approach [4]. A lack of clinical specificity, which can lead to false alerts and undetected events is considered the major weakness of syndromic surveillance [5]. The major advantages are timeliness, flexibility in using the approach for different types of health threats, and cost-effectiveness, because no additional data have to be collected [6, 7].

There are frameworks for evaluating syndromic surveillance systems, which propose a range of quantitative performance indicators such as timeliness and validity, and more qualitative indicators such as flexibility and acceptability [8, 9]. Evaluations to date mostly focus only on single, usually quantifiable performance indicators [10–13]. This is likely due to lack of data or the effort to collect data for all the different performance indicators. Further, there are many case reports assessing one syndromic surveillance system at a time. There are only few comparative analyses or syntheses of several syndromic surveillance systems allowing for a generalizable assessment [7, 11, 14]. Such comparisons could unveil differences in performance and the impact of certain system characteristics on performance. Decision makers could use this information to design syndromic surveillance systems and improve their performance. Such comparative analyses can become a complex endeavour because different characteristics of a syndromic surveillance system can affect performance. These can be the analysed data source, the data collection, analysis and reporting process, and the purpose for and context in which the system is set up. The social science method Qualitative Comparative Analysis (QCA) offers the opportunity to analyse a combination of different system characteristics related to certain performance measures by analysing several case reports at the same time. We aimed at applying QCA for evaluating performance and identifying general success factors of syndromic surveillance systems in Europe.

## Materials and Methods

### Qualitative Comparative Analysis

The aim of QCA is to identify if certain conditions or combinations of conditions (so called configurations, in our case characteristics of syndromic surveillance systems) are part of an outcome set (in our case defined by the performance of syndromic surveillance systems). QCA is an approach and analysis method based on Boolean algebra. It allows for a systematic comparative analysis of small-N and especially case-based data which are not suited for statistical analysis such as regression analysis [15]. Ragin gives a good introduction to QCA in a short online presentation [16]. Until now, QCA was mainly applied in the social sciences, especially the political sciences. It is relatively new to the health sciences. Few studies used it to identify determinants of health policies or interventions but not for analysing topics in public health surveillance [17–19].

QCA allows for analysing quantitative, qualitative or mixed data across several cases (in our case reports of syndromic surveillance system applications for a specific purpose). Depending on the characteristics of the data input, one can choose between two main QCA variants. Crisp set QCA (cs/QSA) analyses dichotomous data while fuzzy set QCA analyses ordinal or interval data or ratios. QCA is an iterative process, characterised by a dialogue between the results of the different steps of the QCA and the researcher’s case knowledge and expertise. The data input for the QCA is not fixed a priori but is likely to be adjusted in the process to increase validity of the results. Nevertheless, QCA follows a structured approach of predefined steps: (1) building a data table consisting of outcome indicators and conditions, (2) constructing a “truth table” consisting of configurations (combinations of the conditions and the outcome), (3) Boolean minimisation to reduce the complexity of the configurations to necessary or sufficient configurations known as solution terms, and (4) interpretation of the solution terms [20]. A key aspect of QCA is the inclusion of configurations without an observed outcome, so called logical remainders [20]. Thus, QCA does not only analyse the observed cases. It includes all logical possible configurations in the minimisation process to reduce the complexity of the solution terms. We found that cs/QCA is the suitable analysis method for our study because of our small-N, case-based and mixed data basis reflecting absence or presence of case characteristics. In the following, we present our setup of the QCA steps 1 to 3.

### Data table: conceptual model

There are two main advantages of syndromic surveillance: it provides timelier information and information on events for which no other public health information is available. These virtues are mainly applied for two purposes: (1) timelier detection of seasonal infectious disease outbreaks, mainly influenza, and (2) real-time situational awareness during events with potential public health impact such as environmental threats or mass gatherings. The latter also includes reassurance that an event has no public health impact. We performed two cs/QCA to identify success factors of syndromic surveillance systems for these two major areas of application.

### Data table: data basis

The data for this study was collected in the framework of the European project Triple S-AGE, which aimed at supporting a harmonised setup of syndromic surveillance systems across Europe [21]. Based on a literature review and an inventory, 60 European syndromic surveillance systems were identified [2, 22]. 36 systems in eight countries and one European consortium were selected for a site visit based on the extent of syndromic surveillance experience. Active, pilot, planned and expired syndromic surveillance systems were visited during nine site visits between June 2011 and June 2012. Data on the visited syndromic surveillance systems were collected through presentations and transcripts of discussions [23]. The 18 syndromic surveillance systems initially selected for this study were chosen based on two aspects: (1) the status of the system, that is if it is or was active and is not just a pilot or planned system AND (2) sufficient availability of data for the study, defined as results published in peer-reviewed journals and coverage during the site visits (Table 1). In February 2015, we updated the literature review purposefully on the selected systems searching in Google Scholar and PubMed and by hand-searching references (list of publications included in data collection available in S1 Appendix). Case-based data for the QCA were compiled from scientific and grey literature and the site visits. The visits provided additional information for the QCA beyond what was reported in scientific publications. In the course of the QCA, we chose to add another case from Germany to increase the quality of the QCA. This ad-hoc system was not considered for a site visit because the plan for the site visits was already fixed at the time the system was set up. All necessary data on this system were collected from scientific and grey literature [24, 25].

**Table 1:** Key characteristics of syndromic surveillance systems selected for QCA.

Country	Name/description of syndromic surveillance systems	Syndromic surveillance data source
**Denmark**	DMOS surveillance	Primary care
**Denmark**	BioAlarm	Emergency medical dispatch centre
**England**	EDSSS	Emergency department
**England**	OOH/Unscheduled care surveillance system	Primary care
**England**	QSurveillance	Primary care
**England**	NHS Direct	Telephone helpline
**France**	SurSaUD—OSCOUR	Emergency department
**France**	SurSaUD—SOS Médecins	Primary care
**Germany**	O104:H4 outbreak surveillance	Emergency department
**Italy**	Migrant influx surveillance	Health services at migrant centre
**Italy**	National emergency department surveillance	Emergency department
**Italy**	Genoa syndromic surveillance system	Emergency department
**Italy**	Lazio Region syndromic surveillance system	Emergency department
**Scotland**	NHS24	Telephone helpline
**Scotland**	PiPeR / SISRS	Primary care
**SIDARTHa**^a^	SIDARTHa-Cantabria	Emergency department
**SIDARTHa**^a^	SIDARTHa-Tyrol	Emergency medical dispatch centre
**Sweden**	GETWELL	Web queries
**Sweden**	1177 telephone helpline surveillance	Telephone helpline

^a^ SIDARTHa = European syndromic surveillance initiative currently comprising two active systems in Cantabria/Spain and Tyrol/Austria

### Data table: outcome indicators

#### Syndromic influenza surveillance

We anticipate a syndromic surveillance system to detect cases earlier if it analyses data that is collected earlier in the course of illness compared to data analysed by traditional surveillance systems. Further, we anticipate that a syndromic surveillance system detects cases earlier if it is based on pre-diagnostic clinical data such as chief complaints, in comparison to confirmed diagnoses [2, 11, 26]. The data basis was largest for seasonal influenza surveillance. Therefore, we focused on this area of application in the QCA. We had to define a cut-off for differentiating a successful from an unsuccessful case in the QCA. We chose this based on the data reported in the cases and based on the following theoretic consideration. Traditional influenza surveillance information based on sentinel general practitioner reports or laboratory confirmations is usually available on a weekly basis. We defined a successful case as a syndromic surveillance system indicating the onset or peak of the influenza season the week before a traditional surveillance system first indicated the same. We used the average timeliness per system if results were reported for several influenza seasons. The outcome indicator for the QCA (QCA coding: OUTCOME) was coded as 1 for successful cases if timeliness of case detection was less than 0 weeks. The outcome was coded as 0 for unsuccessful cases if the timeliness of case detection was 0 or more weeks. Data on syndromic influenza surveillance were available for nine syndromic surveillance systems in six countries (Table 2).

**Table 2:** Raw data for cs/QCA of seasonal syndromic influenza surveillance.

Cases / Systems	Country	NONCLIN	ACUTE	SUBNAT	AGE	Timeliness of detection [weeks]	OUTCOME
1177	SE	1	0	0	0	-0.9	1
GETWELL	SE	1	0	0	0	-1.5	1
Genoa	IT	0	1	1	1	-1.9	1
SIDARTHa Cantabria	SID	0	1	1	0	-0.5	1
NHS24	SC	1	0	0	0	-1.0	1
NHSDirect	EN	1	0	1	1	-0.5	1
QSurveillance	EN	0	0	1	0	2.0	0
Oscour	FR	0	1	0	0	0,0	0
SOS Medecins	FR	0	1	1	0	-2.5	1

0 = absent, 1 = present, ACUTE = acute care data sources, AGE = age-group analysis, EN = England, FR = France, IT = Italy, NONCLIN = non-clinical data sources, SC = Scotland, SE = Sweden, SID = SIDARTHa system, SUBNAT = subnational analysis

#### Situational awareness

We consider syndromic situational awareness as beneficial if a syndromic surveillance system can be applied to different potential public health threats (applicability) and if it provides rapid information (timeliness of reporting) [7, 27, 28]. We defined the cut-off for a successful outcome for applicability as prospective surveillance during two or more types of events. Four different event types can be differentiated: (1) environmental threats such as the volcanic ash plume 2010, heat waves, or floods, (2) the A/H1N1 pandemic 2009, (3) mass gatherings such as political summits or sporting events, and (4) industrial accidents. We chose the definition of this outcome indicator and its cut-off based on the reported applications in the cases. We combined the applicability outcome indicator with an indicator for timeliness. Here, we defined successful outcome as reporting of syndromic surveillance results of under 3 days. Reporting time referred to the onset of the health impact of an event or to the frequency of reporting. We used the average timeliness if data were reported for several events. Also this cut-off was derived based on the reported applications in the cases. If the two outcome indicators contradicted each other, we decided to weigh applicability higher than timeliness. As can be seen from the cases, most syndromic surveillance systems provide timely reports but it seems more difficult to apply a system to more than one type of event. Therefore, the outcome indicator for the QCA (OUTCOME) was coded as 1 for successful cases if the timeliness of reporting was under 3 days and/or the system was applied during two or more types of events. The outcome was coded as 0 for unsuccessful cases if timeliness was 3 or more days and/or the system was applied during only one type of event. Data on situational awareness were available on syndromic surveillance systems in nine countries (Table 3).

**Table 3:** Raw data for cs/QCA of syndromic situational awareness.

Cases	Events (Systems)	MULT-DATA	MULT-SYND	FREE-TEXT	AUTOM	EXIST	ELEC	Applica-bility [no. events]	Timeliness of reporting [days]	OUT-COME
DE	O104:H4 (ad-hoc system)	0	0	0	0	0	0	1	2	0
DK	Pandemic (DMOS)	0	0	0	1	1	1	1	2	0
EN	Various (EDSSS, OOH, Qsurv, NHSDirect)	1	1	0	1	1	1	4	2	1
FR	Various (Oscour, SOS Medecins)	1	1	0	1	1	1	4	0.63	1
IT	Migrant influx (ad-hoc system)	0	1	0	0	0	0	1	4.41	0
SC	Various (different, mainly NHS 24)	1	1	1	1	1	1	3	0.91	1
SE	Pandemic, Volcanic ash plume (GETWELL)	0	1	1	1	1	1	2	2	1
SID	Volcanic ash plume, pandemic (Tyrol/Austria, Cantabria/Spain)	1	1	0	0	1	1	1	14	0

0 = absent, 1 = present, AUTOM = automated syndromic surveillance system components, DE = Germany, DK = Denmark, ELEC = electronic data collection, EN = England, EXIST = syndromic surveillance system existed before monitored event, FR = France, FREETEXT = free text analysis, IT = Italy, MULTDATA = multiple data sources, MULTSYND = multiple syndromes, SC = Scotland, SE = Sweden, SID = SIDARTHa

### Data table: conditions

#### Syndromic influenza surveillance

Timeliness of seasonal influenza case detection can be influenced by the analysed data source. We expect non-clinical data sources (NONCLIN) and information collected prior to confirmed diagnoses from acute care data sources (ACUTE) to support timelier case detection. Non-clinical data sources comprised web searches and telephone helplines. Clinical sources referred to primary and acute care data sources. Acute care data sources were referring to emergency departments or out-of-ours general practitioner services. After a first cs/QCA analysis of these two conditions, a contradictory configuration occurred. That means that the same combination of conditions resulted in a positive and a negative outcome in the observed cases. The most frequently chosen action to resolve this contradiction is to add conditions [20]. We chose the analysis of population subgroups as additional condition. The analysis of different age groups can support timeliness of detecting cases (AGE). Furthermore, the analysis of small-scale, subnational syndromic surveillance data can support timeliness in comparison to traditional surveillance data referring to a higher administrative level (SUBNAT).

#### Situational awareness

Factors influencing applicability of syndromic surveillance systems for various events are flexibility and acceptance of the system. A common experience shared during the site visits was that flexibility is supported by the analysis of multiple data sources (MULTDATA). We added the ability to generate multiple syndromes as another condition (MULTSYND). This was a logical deduction from the analysis of multiple data sources. Furthermore, free text in comparison to coded diagnostic information was reported as supporting factor (FREETEXT) [24]. Acceptability and timeliness of the system is supported by the collection of electronic compared to paper-based data (ELEC). We chose automation of data transfer and possibly data analysis and reporting as additional condition (AUTOM). Furthermore, the existence of a system before the occurrence of the event compared to systems that are set up ad-hoc during an event can support acceptability and timeliness of reporting (EXIST).

### Quality of the data table

A limited variety and consistency can reduce the quality and informational value of the cs/QCA results [20]. In order to check the quality of the data table for the minimisation process, we analysed the variety of values across outcome, conditions and cases. Further, we analysed the consistency of the conditions for explaining a positive outcome. As a rule, there should be a mix of cases with a negative and a positive outcome. Furthermore, at least one third of the cases should represent a certain condition value. And, conditions should not have the same values across the cases. There are no rules for defining appropriate levels of consistency; this depends on the study. But, there is a general agreement about the lower level of consistency of 0.75 [29]. To increase quality of the data table, the selection of cases and conditions or the definition of conditions can be reconsidered [20].

#### Syndromic influenza surveillance

The condition AGE did not show enough variety across all cases and we excluded it from the minimisation process. The consistency levels of the three conditions left for the minimisation process were between 0.43 and 0.57 (Table 4). There is a limitation to take into account when analysing the condition ACUTE. Only clinical data sources can also be acute data sources. The consistency level for ACUTE is rising to 0.75 when referring only to cases using clinical data sources instead of all cases. Despite the low consistency levels for the conditions NONCLIN and SUBNAT, we included the conditions in the analysis. We took the low consistency for these conditions into account in the interpretation of the results.

**Table 4:** Truth table for cs/QCA of seasonal syndromic influenza surveillance.

NONCLIN	ACUTE	SUBNAT	OUTCOME	Cases
1	0	0	1	1177,GETWELL,NHS24
0	1	1	1	GENOA, SID CANT,SOSMEDECINS
1	0	1	1	NHSDIRECT
0	0	1	0	QSURVEILLANCE
0	1	0	0	OSCOUR
0.57	0.43 (0.75)	0.57		Consistency (consistency only for cases NONCLIN = 0)

0 = absent, 1 = present, ACUTE = acute care data sources, NONCLIN = non-clinical data sources, SUBNAT = subnational analysis

#### Situational awareness

For three conditions, MULTSYND, EXIST and ELEC, there was not enough variety over all cases and the conditions ELEC and EXIST were showing the same value pattern over all cases. We decided to exclude the condition ELEC from the minimisation process. Data are more and more electronically available. Thus, this factor will be of lesser relevance in the future compared to the factor of existence before an event. For increasing the variety of values across cases, we had no theoretic justification to exclude any of the cases or conditions or to reconsider the coding of the conditions. Instead, we decided to add another case. We chose the system from Germany, which was set up to monitor the O104:H4 outbreak in 2011. We have analysed this syndromic surveillance system in the framework of another study [28]. We knew that it was qualifying for the QCA and that we could retrieve all necessary data from the broad publication coverage of this case. The consistency levels of the conditions were between 0.75 and 1.00, except for FREETEXT for which it was only 0.5. Thus, we decided to exclude FREETEXT from the minimisation process (Table 5).

**Table 5:** Truth table for cs/QCA of syndromic situational awareness.

MULTIDATA	MULTISYND	AUTOM	EXIST	OUTCOME	Cases
0	0	1	1	0	Denmark
1	1	1	1	1	England, France, Scotland
0	1	0	0	0	Italy Migrants
1	1	0	1	0	SIDARTHa
0	1	1	1	1	Sweden
0	0	0	0	0	Germany
0.75	1.00	1.00	1.00		Consistency

0 = absent, 1 = present, AUTOM = automated syndromic surveillance system components, EXIST = syndromic surveillance system existed before monitored event, MULTDATA = multiple data sources, MULTSYND = multiple syndromes,

### Truth table and Boolean minimisation

We used the software TOSMANA Version 1.302 for accomplishing the cs/QCA steps of constructing the truth table and Boolean minimisation [30].

#### Syndromic influenza surveillance

The truth table contained five configurations of the three conditions (Table 4). Six cases were combined into two groups of configurations while the other three had individual configurations. The first round of Boolean minimisation including logical remainders was based on contradictory simplifying assumptions. That means that a logical remainder was used to explain both positive and negative outcome at the same time. This was resolved by applying the following procedure suggested by Delreux and Hesters [31]. The contradictory simplifying assumption was assigned the likelier outcome of 1 according to case and theoretic knowledge. It was included as logical remainder in the minimisation process for successful cases. The contradictory simplifying assumption was excluded as logical remainder from the minimisation process of the less likely outcome of 0. Instead, it was included as additional case.

#### Situational awareness

The truth table contained six configurations of the five conditions (Table 5). Three cases were combined in one configuration group while the other cases had individual configurations. The inclusion of logical remainders in the minimisation process did not result in any contradictions.

## Results and Discussion

### Results of the Qualitative Comparative Analysis

#### Syndromic influenza surveillance

In more than 75% (n = 7) of the cases, syndromic surveillance systems detected the onset or peak of an influenza season earlier compared to traditional surveillance systems (Table 2). Timeliness ranged from -2.5 weeks in the SOS Medecins system to 2.0 weeks in the QSurveillance system with an average of -0.75 weeks and a median of -0.9 weeks. The minimisation process resulted in two different solution terms explaining each 57% (n = 4) and 43% (n = 3) of the successful cases. Further, it resulted in two solution terms explaining the unsuccessful cases, each explaining 50% (n = 1) of the cases (Table 6). According to these solutions, successful cases of syndromic surveillance systems for timely influenza case detection are analysing non-clinical data sources. Alternatively, they are analysing acute data sources in combination with applying subnational data analysis. In unsuccessful cases, systems are analysing clinical data sources in combination with either analysing non-acute care data or without applying subnational analysis. No solutions leading to successful cases are necessary as the outcome also occurred in the absence of the solutions. The solutions are sufficient as the outcome always occurred when the solutions are present. But, there are also other solutions leading to the outcome.

**Table 6:** Solution terms for explaining successful and unsuccessful cases of seasonal syndromic influenza surveillance.

Solution terms	Cases	RC	UC	SC	C
NONCLIN+	(1177,GETWELL,NHS24+NHSDIRECT)	0.57	0.57		1.0
ACUTE*SUBNAT	(GENOA+SID CANT,SOSMEDECINS)	0.43 (1.0)^a^	0.43 (1.0)^a^		1.0
→ OUTCOME				1.0	1.0
nonclin*acute	(QSURVEILLANCE)	0.5 (0.5) ^a^	0.5 (0.5) ^a^		1.0
nonclin*subnat	(OSCOUR)	0.5	0.5		1.0
→ outcome				1.0	1.0

Capital letter = presence, small letter = absence, + = logical OR, * = logical AND, → = sufficient relation, ACUTE = acute care data sources, C = consistency, NONCLIN/nonclin = non-clinical data sources, RC = raw coverage (number of cases covered by solution of all cases with the same outcome), SC = solution coverage (number of cases covered by all solutions of all cases with the same outcome), SUBNAT/subnat = subnational analysis, UC = unique coverage (number of cases uniquely covered by a solution of all cases with the same outcome)

^a^ coverage value in brackets refer only to clinical cases

#### Situational awareness

The systems covered different types of events ranging from one event in four cases to four events in two cases (Table 3). Reporting time for the six successful cases was around one day. The timeliness over all cases was very similar. For the two unsuccessful cases, reporting time was around 4 and 14 days. The minimisation process resulted in one solution term explaining all successful cases. Two solution terms explained 50% (n = 2) and 75% (n = 3) of the unsuccessful cases respectively (Table 7). According to the solutions, in successful cases of situational awareness, syndromic surveillance systems are analysing multiple syndromes and are automated. In unsuccessful cases, systems are analysing single syndromes and/or are not automated. The combination of analysing multiple syndromes in an automated system is a necessary condition as it was always present when the outcome occurred. Further, the outcome did not occur when this configuration was absent. The analysis of multiple data sources and the existence of the system before the event occurred were not identified as key influencing factors.

**Table 7:** Solution terms for explaining successful and unsuccessful cases of syndromic situational awareness.

Solution terms	Cases	RC	UC	SC	C
MULTSYND*AUTOM	(England,France,Scotland+Sweden)	1.0	1.0		1.0
← OUTCOME				1.0	1.0
multsynd+	(Denmark+Germany)	0.5	0.25		1.0
autom	(Italy Migrants+SIDARTHa+Germany)	0.75	0.5		1.0
→ outcome				1.0	1.0

Capital letter = presence, small letter = absence, + = logical OR, * = logical AND, → = sufficient relation, ← necessary relation, ACUTE = acute care data sources, AUTOM/autom = automated syndromic surveillance system components, C = consistency, MULTSYND/multsynd = multiple syndromes, RC = raw coverage (number of cases covered by solution of all cases with the same outcome), SC = solution coverage (number of cases covered by all solutions of all cases with the same outcome), UC = unique coverage (number of cases uniquely covered by a solution of all cases with the same outcome)

### Interpretation

#### Syndromic influenza surveillance

The low coverage levels of the QCA solution terms reflect the low consistency levels of the data input. They call for careful interpretation of the findings and indicate a limitation of their practical relevance. The solution term for successful cases confirmed our hypothesis that the analysis of non-clinical data can support timely syndromic influenza surveillance. The analysis of acute care data sources seems to be of lesser importance and only in combination with another condition such as subnational analysis.

Only cases featuring clinical data based systems were unsuccessful. In one of the two unsuccessful cases, QSurveillance, the system is based on data from general practitioners. In the course of illness or patient treatment, this data source is positioned closer to the traditional data sources of confirmed diagnoses from sentinel general practitioners or laboratories. Thus, compared to the non-clinical data sources, timeliness of general practitioner data sources must be lower. The timeliness of the second system, Oscour, which is based on emergency department data, was referring to two influenza seasons in 2005 and 2006. Since then, the system was used for influenza surveillance in France on a regular basis. It might very well have shown timeliness values in other seasons that were comparable to the other acute care data based systems that we analysed. We expected subnational data analysis to have an impact on the performance. According to the solution term, the analysis of age groups does not seem to influence timely case detection. That does not mean that age-adjusted analysis does not have an impact on timelier case detection. Such analysis might just not be applied in many systems. QCA says more about conditions in relation to each other than about the impact of single conditions [29].

When looking at applications of the analysed syndromic surveillance systems for other infectious disease outbreaks, the two QCA solutions for successful outcome also apply to those cases. For detection of seasonal gastrointestinal outbreaks, the NHS Direct system in England achieved a timeliness of -5.68 weeks on average using non-clinical data [32]. No results from subnational analysis were reported, but from analysis by age group. The syndromic surveillance system in Genoa monitored two measles outbreaks with a timeliness of -3.57 weeks on average based on acute care data [33, 34]. In this case, subnational surveillance was applied in one study and was connected to a 1.43 week advance detection [34]. No results from age-adjusted analysis were reported. We could only collect data on syndromic surveillance of other infectious disease outbreaks for these two systems. Therefore, these findings can only indicate a confirmation of our results.

Looking into other systems for syndromic seasonal influenza surveillance outside Europe, we can best look into a review by Dailey and colleagues [11]. They compared different systems and data sources for timeliness of syndromic seasonal influenza surveillance. The authors also found a higher timeliness of systems analysing non-clinical and acute care data sources. When looking into the single studies included in the review, there were no comparable non-clinical data based systems. Six systems were based on emergency department data. These are comparable to the acute care data based systems included in our study. One of these systems was associated with a negative outcome according to our definition. This system applied subnational analysis [35]. Another system applied subnational analysis and had a positive outcome [36]. Three other systems associated with positive outcome according to our definition did not report application of subnational analysis [37–39]. One applied spatial cluster analysis but did not find any clusters associated with influenza [40]. One also applied age-adjusted analysis [39]. From the six systems, five only reported results for one influenza season, which reduces the representativeness of the results [35–37, 39, 40]. We included cases of systems analysing emergency department data, which were all based on more than one season. The sixth study did not compare syndromic with traditional surveillance results, which limits the comparability to our results [38].

The comparison with other systems indicates that the two conditions concerning data sources might influence timeliness of syndromic influenza surveillance. The analysis of non-clinical data sources might have a greater positive influence than the analysis of acute data sources. Subnational analysis in combination with acute care data sources seems to be of lesser influence.

#### Situational awareness

The possibility to analyse multiple syndromes and the use of automated systems were identified as key success factors, which is in line with our hypothesis. Even more, the combination of these factors turned out to be a necessary configuration for successful cases. This solution term explained all observed successful cases. The absence of one or both of these conditions was connected with unsuccessful cases of syndromic situational awareness. Against the expectations formulated during the site visits, the condition of analysing multiple data sources turned out not to be a success factor for flexible situational awareness. Multiple data sources were analysed in successful and unsuccessful cases. Further, in successful and unsuccessful cases, systems existed before the monitored event. However, in all successful cases, systems were established before the event. This indicates that this aspect could support syndromic situational awareness.

Systems outside Europe, which were only applied during one event, shared similar characteristics with the systems labelled as unsuccessful cases in our study. One example are systems that were set up in 2005 in the aftermath of hurricane Katrina in different cities in the United States of America. They analysed multiple databases and multiple syndromes on a daily basis. But, they were not existing before the event, and were based on manual data collection and analysis [41, 42]. Another example is the system set up for monitoring the Kentucky Derby in Louisville from 2002 until 2004. The system was neither automated nor established prior to the event (and terminated between events). But, it was monitoring multiple syndromes on a daily basis [43]. Also in these examples, the combination of multiple syndrome analysis and automation was absent as suggested by the QCA solution.

Turning to successful cases of systems outside Europe, we can look at the system in New York City, which analysed different types of events. Next to multiple other data sources, especially emergency department data were used for situational awareness [44]. The emergency department data based system part was set up ad-hoc as non-automated system in the days after the September 2011 terrorist attacks [45, 46]. The system was maintained by changing it into an automated system. The automated emergency department data based system was used to monitor multiple syndromes during the blackout in 2003 [47], the A/H1N1 pandemic 2009 [48, 49] and the effects of hurricane Sandy in 2012 [50]. Another system part, which was analysing multiple other data sources, was only used for monitoring one syndrome during the blackout in 2003 [47]. This system part seemed to be automated for certain data sources, but the information we could obtain from the literature was unambiguous. For at least one part of the New York City system, the combination of multiple syndrome analysis in an automated system supported situational awareness. This example also confirms that the analysis of multiple data sources is not a key success factor.

### Limitations

The low quality of the data table and solution terms of the QCA regarding syndromic influenza surveillance limits the quality and practical relevance of the findings. We tried to include representative data providing results for more than one influenza season. We decided to include cases of two systems despite results referred only to one season: NHS24 in Scotland and QSurveillance in England. NHS24 was applied in more than one influenza season and reported timeliness was positive but not explicitly quantified [51]. As QSurveillance was the only case representing a primary care data based system, we decided to include it to add variety to the QCA. The QSurveillance system was reportedly changed, so performance could also have changed [52]. Nevertheless, the QCA results might not be representative for other syndromic surveillance systems based on general practitioner data.

The conditions analysed in the QCA were chosen based on theoretic considerations but also based on the available data. There are other conditions such as validity or representativeness, which might alter our QCA results. But, we could not include them due to limited data availability, or in the case of validity, because of the large diversity in measuring this indicator in different surveillance systems.

QCA makes it necessary to differentiate successful from unsuccessful cases. We defined the cut-off points for both analyses based on the results reported for the cases and theoretic considerations. We could only include few unsuccessful cases with a limited representativeness in our QCA on syndromic influenza surveillance. This might have limited our options to define a representative cut-off. It is important to highlight that the distinction of successful or unsuccessful cases should not be generalised to the syndromic surveillance systems behind these cases. The syndromic surveillance systems might perform differently when they are monitoring other syndromes, for example.

Finally, the data in our study were not collected with QCA in mind. We chose the method afterwards to add value to our complex case-based data. With QCA in mind, we might have collected different or additional data, which could have improved quality of the input and output of the QCA.

### Practical relevance

For the first time, QCA allowed a structured, comparative analysis of complex, small-N case reports of syndromic surveillance system performance. The identified success factors can inform the process of designing or further developing a syndromic surveillance system for situational awareness or seasonal influenza surveillance. The success factors could be used to prioritise certain system characteristics over others for improving performance. This especially applies to the system characteristics included in our analysis. For example, a syndromic influenza surveillance system might detect cases earlier rather by analysing non-clinical data than clinical data. Further, the choice of data source seems to influence timely influenza surveillance more than the analysis of subnational level data or age groups. Timely syndromic situational awareness during different types of events is supported by analysing multiple syndromes in an automated system. This combination of system characteristics is of greater influence than the analysis of multiple data sources or the existence of the syndromic surveillance system before the occurrence of an event.

The relatively low coverage levels of the QCA results suggest that the success factors regarding syndromic influenza surveillance should be applied with caution. The relatively high coverage level of the QCA results for syndromic situational awareness suggests a higher reliability of these findings. Any changes in specific syndromic surveillance systems should be based on comprehensive test runs using historical data.

## Conclusions

We identified key success factors for the two main areas of syndromic surveillance system application using cs/QCA. For syndromic influenza surveillance, a system might be timelier if analysing non-clinical data sources. Syndromic situational awareness is supported by automated syndromic surveillance systems capable of analysing multiple syndromes. Analysing multiple data sources is no pre-requisite for flexible situational awareness. We suggest to consider the success factors when designing or further developing a syndromic surveillance system.

We showed that the social science analysis method QCA can add value for the interpretation of case-based, small-N and mixed data in the area of public health surveillance. QCA can only produce valid results if guided by good case and subject expertise and if based on varied and representative data. We propose to apply QCA to other case-based and small-N data next to more traditional analysis methods. It might yield meaningful and relevant results for policy and practice that would otherwise remain undiscovered.

## Supporting Information

S1 Appendix List of publications included in data collection. (PDF)
