# Supplementary material for: Success Factors of European Syndromic Surveillance Systems: A Worked Example of Applying Qualitative Comparative Analysis
Source: PLoS One. 2016 May 16;11(5):e0155535. doi: 10.1371/journal.pone.0155535 (PMC4868285; doi:10.1371/journal.pone.0155535)
Supplement: S1 Appendix — (PDF) [file pone.0155535.s001.pdf]

**Appendix S1**

**List of publications included in data collection**

Andersson T, Bjelkmar P, Hulth A, Lindh J, Stenmark S, Widerstrom M. Syndromic surveillance for local outbreak detection and awareness: evaluating outbreak signals of acute gastroenteritis in telephone triage, web-based queries and over-the-counter pharmacy sales. Epidemiol Infect. 2014;142(2):303-13.

Ansaldi F, Orsi A, Altomonte F, Bertone G, Parodi V, Carloni R, et al. Emergency department syndromic surveillance system for early detection of 5 syndromes: a pilot project in a reference teaching hospital in Genoa, Italy. J Prev Med Hyg. 2008;49(4):131-5.

Ansaldi F, Orsi A, Altomonte F, Bertone G, Parodi V, Carloni R, et al. Syndrome surveillance and molecular epidemiology for early detection and tracing of an outbreak of measles in Liguria, Italy. J Med Virol. 2009;81(10):1807-13.

Baker M, Smith GE, Cooper D, Verlander NQ, Chinemana F, Cotterill S, et al. Early warning and NHS Direct: a role in community surveillance? J Public Health Med. 2003;25(4):362-8.

Bork KH, Klein BM, Molbak K, Trautner S, Pedersen UB, Heegaard E. Surveillance of ambulance dispatch data as a tool for early warning. Euro Surveill. 2006;11(12):229-33.

Cooper DL, Smith G, Baker M, Chinemana F, Verlander N, Gerard E, et al. National symptom surveillance using calls to a telephone health advice service--United Kingdom, December 2001-February 2003. MMWR Morb Mortal Wkly Rep. 2004;53 Suppl:179-83.

Cooper DL, Smith GE, Edmunds WJ, Joseph C, Gerard E, George RC. The contribution of respiratory pathogens to the seasonality of NHS Direct calls. J Infect. 2007;55(3):240-8.

Cooper DL, Smith GE, O'Brien SJ, Hollyoak VA, Baker M. What can analysis of calls to NHS direct tell us about the epidemiology of gastrointestinal infections in the community? J Infect. 2003;46(2):101-5.

Cooper DL, Smith GE, Regan M, Large S, Groenewegen PP. Tracking the spatial diffusion of influenza and norovirus using telehealth data: a spatiotemporal analysis of syndromic data. BMC Med. 2008;6:16. doi:10.1186/1741-7015-6-16.

Cooper DL, Verlander NQ, Elliot AJ, Joseph CA, Smith GE. Can syndromic thresholds provide early warning of national influenza outbreaks? J Public Health (Oxf). 2009;31(1):17-25.

Cooper DL, Verlander NQ, Smith GE, Charlett A, Gerard E, Willocks L, et al. Can syndromic surveillance data detect local outbreaks of communicable disease? A model using a historical cryptosporidiosis outbreak. Epidemiol Infect. 2006;134(1):13-20.

De Florentiis D, Parodi V, Orsi A, Rossi A, Altomonte F, Canepa P, et al. Impact of influenza during the post-pandemic season: epidemiological picture from syndromic and virological surveillance. J Prev Med Hyg. 2011;52(3):134-6.

Doroshenko A, Cooper D, Smith G, Gerard E, Chinemana F, Verlander N, et al. Evaluation of syndromic surveillance based on National Health Service Direct derived data--England and Wales. MMWR Morb Mortal Wkly Rep. 2005;54 Suppl:117-22.

Elliot A. Syndromic surveillance: the next phase of public health monitoring during the H1N1 influenza pandemic? Euro Surveill. 2009;14(44). pii: 19391.

Elliot AJ, Bone A, Morbey R, Hughes HE, Harcourt S, Smith S, et al. Using real-time syndromic surveillance to assess the health impact of the 2013 heatwave in England. Environmental Research. 2014;135:31-6.

Elliot AJ, Hughes HE, Hughes TC, Locker TE, Brown R, Sarran C, et al. The impact of thunderstorm asthma on emergency department attendances across London during July 2013. Emerg Med J. 2014;31(8):675-8.

Elliot AJ, Hughes HE, Hughes TC, Locker TE, Shannon T, Heyworth J, et al. Establishing an emergency department syndromic surveillance system to support the London 2012 Olympic and Paralympic Games. Emerg Med J. 2012;29(12):954-60.

Elliot AJ, Morbey RA, Hughes HE, Harcourt SE, Smith S, Loveridge P, et al. Syndromic surveillance - a public health legacy of the London 2012 Olympic and Paralympic Games. Public Health. 2013;127(8):777-81.

Elliot AJ, Singh N, Loveridge P, Harcourt S, Smith S, Pnaiser R, et al. Syndromic surveillance to assess the potential public health impact of the Icelandic volcanic ash plume across the United Kingdom, April 2010. Euro Surveill. 2010;15(23). pii: 19583.

Flamand C, Larrieu S, Couvy F, Jouves B, Josseran L, Filleul L. Validation of a syndromic surveillance system using a general practitioner house calls network, Bordeaux, France. Euro Surveill. 2008;13(25). pii: 18905.

Gault G, Larrieu S, Durand C, Josseran L, Jouves B, Filleul L. Performance of a syndromic system for influenza based on the activity of general practitioners, France. J Public Health (Oxf). 2009;31(2):286-92.

Green HK, Zhao H, Boddington NL, Andrews N, Durnall H, Elliot AJ, et al. Detection of varying influenza circulation within England in 2012/13: informing antiviral prescription and public health response. J Public Health (Oxf). 2015;37(2):295-304.

Guasticchi G, Giorgi Rossi P, Lori G, Genio S, Biagetti F, Gabriele S, et al. Syndromic surveillance: sensitivity and positive predictive value of the case definitions. Epidemiol Infect. 2009;137(5):662-71.

Guasticchi G, Rossi PG, Lori G, Genio S, Gabriele S, Borgia P. The emergency visit-based syndromic surveillance of the Lazio region. Results of the pilot phase. Ital J Public Health 2009;6(2):150-5.

Harcourt SE, Fletcher J, Loveridge P, Bains A, Morbey R, Yeates A, et al. Developing a new syndromic surveillance system for the London 2012 Olympic and Paralympic Games. Epidemiol Infect. 2012; 140(12):2152-6.

Harcourt SE, Smith GE, Elliot AJ, Pebody R, Charlett A, Ibbotson S, et al. Use of a large general practice syndromic surveillance system to monitor the progress of the influenza A(H1N1) pandemic 2009 in the UK. Epidemiol Infect. 2012;140(1):100-5.

Harder KM, Andersen PH, Baehr I, Nielsen LP, Ethelberg S, Glismann S, et al. Electronic real-time surveillance for influenza-like illness: experience from the 2009 influenza A(H1N1) pandemic in Denmark. Euro Surveill. 2011;16(3). pii: 19767.

Hughes HE, Morbey R, Hughes TC, Locker TE, Shannon T, Carmichael C, et al. Using an Emergency Department Syndromic Surveillance System to investigate the impact of extreme cold weather events. Public Health. 2014;128(7):628-35.

Hulth A, Rydevik G. Web query-based surveillance in Sweden during the influenza A(H1N1)2009 pandemic, April 2009 to February 2010. Euro Surveill. 2011;16(18). pii: 19856

Hulth A, Rydevik G. GET WELL: an automated surveillance system for gaining new epidemiological knowledge. BMC Public Health. 2011;11:252. doi: 10.1186/1471-2458-11-252

Hulth A, Rydevik G, Linde A. Web queries as a source for syndromic surveillance. PLoS One. 2009;4(2):e4378. doi: 10.1371/journal.pone.0004378

Josseran L, Caillere N, Brun-Ney D, Rottner J, Filleul L, Brucker G, et al. Syndromic surveillance and heat wave morbidity: a pilot study based on emergency departments in France. BMC Med Inform Decis Mak. 2009;9:14. doi: 10.1186/1472-6947-9-14

Josseran L, Fouillet A, Caillere N, Brun-Ney D, Ilef D, Brucker G, et al. Assessment of a syndromic surveillance system based on morbidity data: results from the Oscour network during a heat wave. PLoS One. 2010;5(8):e11984.

Josseran L, Nicolau J, Caillere N, Astagneau P, Brucker G. Syndromic surveillance based on emergency department activity and crude mortality: two examples. Euro Surveill. 2006;11(12):225-9.

Kavanagh K, Robertson C, Murdoch H, Crooks G, McMenamin J. Syndromic surveillance of influenza-like illness in Scotland during the influenza A H1N1v pandemic and beyond. J R Stat Soc a Stat. 2012;175:939-58.

Leonardi GS, Hajat S, Kovats RS, Smith GE, Cooper D, Gerard E. Syndromic surveillance use to detect the early effects of heat-waves: an analysis of NHS direct data in England. Soz Praventivmed. 2006;51(4):194-201.

Loveridge P, Cooper D, Elliot AJ, Harris J, Gray J, Large S, et al. Vomiting calls to NHS Direct provide an early warning of norovirus outbreaks in hospitals. J Hosp Infect. 2010;74(4):385-93.

Ma T, Englund H, Bjelkmar P, Wallensten A, Hulth A. Syndromic surveillance of influenza activity in Sweden: an evaluation of three tools. Epidemiol Infect. 2015;143(11):2390-8.

McCloskey B, Endericks T, Catchpole M, Zambon M, McLauchlin J, Shetty N, et al. London 2012 Olympic and Paralympic Games: public health surveillance and epidemiology. Lancet. 2014;383(9934):2083-9.

Meyer N, McMenamin J, Robertson C, Donaghy M, Allardice G, Cooper D. A multi-data source surveillance system to detect a bioterrorism attack during the G8 Summit in Scotland. Epidemiol Infect. 2008;136(7):876-85.

Molbak K, Widgren K, Jensen KS, Ethelberg S, Andersen PH, Christiansen AH, et al. Burden of illness of the 2009 pandemic of influenza A (H1N1) in Denmark. Vaccine. 2011;29 Suppl 2:B63-9.

Morbey RA, Elliott AJ, Charlett A, Andrews N, Verlander NQ, Ibbotson S, et al. Development and refinement of new statistical methods for enhanced syndromic surveillance during the 2012 Olympic and Paralympic Games. Health informatics journal. 2015;21(2):159-69.

Morbey RA, Elliot AJ, Charlett A, Ibbotson S, Verlander NQ, Leach S, et al. Using public health scenarios to predict the utility of a national syndromic surveillance programme during the 2012 London Olympic and Paralympic Games. Epidemiol Infect. 2014;142(5):984-93.

Napoli C, Riccardo F, Declich S, Dente MG, Pompa MG, Rizzo C, et al. An early warning system based on syndromic surveillance to detect potential health emergencies among migrants: results of a two-year experience in Italy. International journal of environmental research and public health. 2014;11(8):8529-41.

Orsi A, Alicino C, Patria AG, Parodi V, Carloni R, Turello V, et al. Epidemiological and molecular approaches for management of a measles outbreak in Liguria, Italy. J Prev Med Hyg. 2010;51(2):67-72.

Riccardo F, Napoli C, Bella A, Rizzo C, Rota MC, Dente MG, et al. Syndromic surveillance of epidemic-prone diseases in response to an influx of migrants from North Africa to Italy, May to October 2011. Euro Surveill. 2011;16(46). pii: 20016.

Rizzo C, Rota MC, Bella A, Giannitelli S, De Santis S, Nacca G, et al. Response to the 2009 influenza A(H1N1) pandemic in Italy. Euro Surveill. 2010;15(49). pii: 19744.

Robert Koch Institute. Final presentation and evaluation of epidemiological findings in the EHEC O104:H4 Outbreak, Germany 2011. Berlin: Robert Koch Institute; 2011.

Rosenkötter N, Ziemann A, Riesgo LG, Gillet JB, Vergeiner G, Krafft T, et al. Validity and timeliness of syndromic influenza surveillance during the autumn/winter wave of A(H1N1) influenza 2009: results of emergency medical dispatch, ambulance and emergency department data from three European regions. BMC Public Health. 2013;13(1):905. doi: 10.1186/1471-2458-13-905

Rosenkötter N, Ziemann, A., Garcia-Castrillo Riesgo, L., Vergeiner, G., Fischer, M., Krafft, T., Brand, H., Lippert, F., Krämer, A., Pinheiro, P., on behalf of the SIDARTHa project group. SIDARTHa Volcanic Ash Cloud Rapid Public Health Impact Assessment. Regional public health impact of volcanic ash cloud covering Europe after eruption of Eyjafjallajoekull, Iceland starting April 14th, 2010. Results as of May 15th, 2010. Bad Honnef: SIDARTHa Scientific-Technical Coordination Office, 2010.

Schrell S, Ziemann A, Garcia-Castrillo Riesgo L, Rosenkotter N, Llorca J, Popa D, et al. Local implementation of a syndromic influenza surveillance system using emergency department data in Santander, Spain. J Public Health (Oxf). 2013;35(3):397-403.

Severi E, Heinsbroek E, Watson C, Catchpole M. Infectious disease surveillance for the London 2012 Olympic and Paralympic Games. Euro Surveill. 2012;17(31). pii: 20232

Smith GE, Bawa Z, Macklin Y, Morbey R, Dobney A, Vardoulakis S, et al. Using real-time syndromic surveillance systems to help explore the acute impact of the air pollution incident of March/April 2014 in England. Environ Res. 2015;136:500-4.

Smith GE, Cooper DL, Loveridge P, Chinemana F, Gerard E, Verlander N. A national syndromic surveillance system for England and Wales using calls to a telephone helpline. Euro Surveill. 2006;11(12):220-4.

Smith S, Elliot AJ, Mallaghan C, Modha D, Hippisley-Cox J, Large S, et al. Value of syndromic surveillance in monitoring a focal waterborne outbreak due to an unusual Cryptosporidium genotype in Northamptonshire, United Kingdom, June - July 2008. Euro Surveill. 2010;15(33). pii: 19643.

Smith S, Smith GE, Olowokure B, Ibbotson S, Foord D, Maguire H, et al. Early spread of the 2009 influenza A(H1N1) pandemic in the United Kingdom--use of local syndromic data, May-August 2009. Euro Surveill. 2011;16(3). pii: 19771.

Timpka T, Spreco A, Dahlstrom O, Eriksson O, Gursky E, Ekberg J, et al. Performance of eHealth data sources in local influenza surveillance: a 5-year open cohort study. J Med Internet Res. 2014;16(4):e116. doi: 10.2196/jmir.3099

Timpka T, Spreco A, Eriksson O, Dahlstrom O, Gursky EA, Stromgren M, et al. Predictive performance of telenursing complaints in influenza surveillance: a prospective cohort study in Sweden. Euro Surveill. 2014;19(46). pii: 20966

Wadl M, Rieck T, Nachtnebel M, Greutelaers B, an der Heiden M, Altmann D, et al. Enhanced surveillance during a large outbreak of bloody diarrhoea and haemolytic uraemic syndrome caused by Shiga toxin/verotoxin-producing Escherichia coli in Germany, May to June 2011. Euro Surveill. 2011;16(24). pii: 19893

Ziemann A, Rosenkötter N, Garcia-Castrillo Riesgo L, Schrell S, Kauhl B, Vergeiner G et al. A concept for routine emergency care data based syndromic surveillance in Europe. Epidemiol Infect. 2014;142(11):2433-46.
